# EpiLog: A software for the logical modelling of epithelial dynamics

**DOI:** 10.12688/f1000research.15613.2

**Published:** 2019-03-11

**Authors:** Pedro L. Varela, Camila V. Ramos, Pedro T. Monteiro, Claudine Chaouiya

**Affiliations:** 1 Instituto Superior Técnico, Universidade de Lisboa, Lisbon, P-1049-001, Portugal; 2 INESC-ID, Lisbon, P-1000-029, Portugal; 3 Instituto Gulbenkian de Ciência, Oeiras, P-2780-156, Portugal; 4 CNRS, Centrale Marseille, l'Institut de Mathématiques de Marseille, Aix-Marseille University, Marseille, France

**Keywords:** Logical modelling, Multicellular regulatory networks, Cellular automaton, Hexagonal grid

## Abstract

Cellular responses are governed by regulatory networks subject to external signals from surrounding cells and to other micro-environmental cues. The logical (Boolean or multi-valued)  framework proved well suited to study such processes at the cellular level, by specifying qualitative models of involved signalling pathways and gene regulatory networks.

Here, we describe and illustrate the main features of EpiLog, a computational tool that implements an extension of the logical framework to the tissue level. EpiLog defines a collection of hexagonal cells over a 2D grid, which embodies a mono-layer epithelium. Basically, it defines a cellular automaton in which cell behaviours are driven by associated logical models subject to external signals.

EpiLog is freely available on the web at http://epilog-tool.org. It is implemented in Java (version ≥1.7 required) and the source code is provided at https://github.com/epilog-tool/epilog under a GNU General Public License v3.0.

## Introduction

Pattern formation emerges from the interplay of interaction networks, at the cellular and multi-cellular levels ^1^. To uncover the complex mechanisms at stake, computational modelling is very needed. In this context, cellular automaton approaches are a natural choice of modelling framework, particularly well suited to define local rules governing the behaviours of cells over a lattice ^2^. On the other hand, the logical formalism has proved efficient to explore cellular regulatory networks driving developmental processes (for a recent review on the logical modelling approach, see 3). However, the consideration of ensembles of communicating cells is often required to recapitulate observed patterns (e.g. 4, 5). This motivated the development of EpiLog, which implements an extension of the logical framework to hexagonal grids embodying simple epithelia.

Briefly, a logical model assigns discrete (Boolean or multi-valued) variables to the regulatory components, and logical regulatory rules define the evolution of the variables depending on the variables associated with the component regulators. Input components embody cell receptors receiving external signals. Unlike internal components, inputs have no associated regulatory rules, and are generally maintained at constant values accounting for fixed environmental conditions. A model state is given by the values of the components, and the dynamics is generated according to specific updating schemes. In a synchronous update, a state has at most one successor state in which all the variables have been updated as prescribed by the regulatory rules. In contrast, the asynchronous update generates as many successors as the number of updated variables, yielding more complex and non-deterministic behaviours. Properties of the resulting dynamics relate to the model attractors (stable states or point attractors, and cyclical attractors), which can be associated with different phenotypes. Beyond identifying the attractors, a major question relates to reachability properties: given an initial condition, what is (are) the reachable attractor(s)? The definition, analysis and simulation of logical models of cellular networks can be performed using one of the existing software tools ^6, 7^. Many of these support the exchange format SBML qual (Qualitative Models package for SBML) ^8, 7^.

To handle multi-cellular systems, Mendes *et al.* have established a compositional approach relying on a process algebra framework to assess stable states reachability in asynchronous dynamics ^9^. This approach showed some limitations due to the huge size of the considered state spaces, and is thus limited to the analysis of models encompassing a reduced number of cells (up to a dozen cells). In contrast to the previous compositional approach, EpiLog relies on a cellular automata framework, which allows to scale model simulations up to thousands of cells. Still, cell-cell interactions are specified using (logical) integration rules, in a way similar to that proposed by Mendes *et al.* ^9^. These rules govern the evolution of cellular input components, depending on the values of internal components (representing e.g. secreted proteins) in neighbouring cells.

Multiple simulation platforms have been developed to support the modelling of multi-cellular systems. A detailed overview of existing approaches and application domains is beyond the scope of this paper. To our knowledge, only two tools exist that integrate Boolean models in a multi-cellular context. The first, named CoGNaC ^10^, is a Chaste plugin. Chaste is an open-source simulation package that includes tissue modelling. As such, it supports distinct cell-based modelling approaches ^11, 12^. The second tool, named PhysiBoSS ^13^, combines intra-cellular Boolean signalling networks as handled by MaBoSS ^14^ and multi-cellular agent based modelling as defined in PhysiCell, an open source agent-based simulator ^15^. While these tools involve explicit representations of intra-cellular networks in the form of Boolean models, and the consideration of biophysical processes governing multi-cellular behaviours, they do not allow explicit specification of cell-cell communication, a special feature of EpiLog.

## Methods

### Implementation

EpiLog is implemented in Java (requires Java 7 or higher), is freely available at http://epilog-tool.org, and launches a Graphical User Interface (GUI) based on JFC/Swing. It is provided as a single .jar file which can be launched from a graphical file manager by double clicking on it or through the command line as follows:



                        $ java -jar EpiLog-versionX.jar [--file /path/to/multicellular_project.peps]
                    


where the flag --file can be used to optionally specify the file to be open. EpiLog .peps files represent projects containing multiple multicellular models, each with potentially distinct characteristics.

Additionally, an EpiLog model repository is provided at http://epilog-tool.org/models_repository where users can deposit their models by contacting support@epilog-tool.org. Each model page includes a title, a description, a taxon and process classification, model .peps file(s) and supporting paper(s).

Developers can freely access the code repository at http://github.com/epilog-tool/epilog, clone it and extend the code. For dependency management of external libraries, EpiLog relies on Apache Maven. A particular library is the bioLQM Java toolkit (Logical Qualitative Models of biological regulatory networks), for the representation and manipulation of logical, cellular models, available at https://github.com/colomoto/bioLQM ^16^.

### Operation

**Model definition** EpiLog defines projects that include multi-cellular models, called *epithelium models*, each being specified by:

1. The size of the hexagonal grid, as well as its border conditions; vertical and horizontal borders can be connected, or not leading to rectangular, cylindrical or toroidal grids;
2. A (set of) logical model(s), each associated with cells of the grid; these *cellular models* can be defined in any tool supporting the SBML qual format ^8^. EpiLog imports SBML files and identifies input components in these cellular models;
3. The cellular input components; these are defined as constant *positional inputs* (e.g. morphogens produced by external sources) or evolving *integration inputs* (e.g. juxtacrine signals, or other secreted molecules influencing cells in the neighbourhood); for the latter, logical *integration rules* specify which signals are received from (internal) components of neighbouring cells and how these signals are combined;
4. An initial state defining the values of the internal components of all the cells.

**Figure 1.**
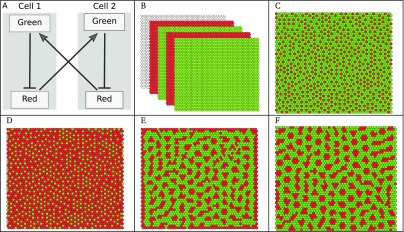
( **A**) An idealised Boolean model of lateral inhibition; the Green marker is induced by the Red marker secreted by the neighbouring cell, whereas Red is induced by low levels of Green in the same cell (normal arrows denote activations, whereas blunt arrows denote inhibitions); the synchronous dynamics of this two cell model leads to a cyclic attractor (see Supplementary File 1). ( **B**) Simulation in EpiLog, with the same cellular model over a square grid of 50×50 hexagonal cells under a synchronous update (Green induced if at least one contacting cell is Red), oscillations are due to the synchronous update of the cells, see Supplementary Movie 1. ( **C**– **F**) Simulations under α-asynchrony (α = 0.25): ( **C**) Stable state of the grid, referred to as “stable pattern”, reached in 29 steps (Green induced if at least one contacting cell is Red), see Supplementary Movie 2; ( **D**) Stable reverted pattern reached in 30 steps (Green induced if all contacting cells are Red); ( **E**) Stable pattern reached in 63 steps (Green induced if at least 12 cells at distance up to 3 are Red); ( **F**) Stable pattern reached in 70 steps, with the same setting as for panel **E** but considering a torus ( *i.e.*, no grid borders).

**Simulation settings** Simulation settings define the updating schemes for both the cellular and epithelium models. The default scheme is synchronous, that is to say, at each iteration, all the cellular models are considered for a synchronous update of their internal components. To overcome spurious oscillations generated by these synchronous updates (see Figure 1A and Supplementary File 1), EpiLog allows to specify:

1. The cellular model update, with the definition of (synchronous) priority classes (increasing or decreasing) updates are gathered in ordered sets, allowing to account for different time scales for the mechanisms underlying updates of internal component ^17^;
2. The epithelium update, with the definition of probabilistic updates of the cell states, inspired by N. Fatès’ alpha-asynchronism ^18, 19^: parameter *α* specifies the proportion of the cells randomly chosen for update (see Supplementary File 2 for an illustration).

**Model perturbations** The logical formalism permits to easily specify perturbations (e.g. knock-out or ectopic expression of cellular model components) by blocking the values of perturbed components. EpiLog includes this feature with the definition of component perturbations that can be applied to all the cells or to restricted regions of the grid.

**Model clones** At any simulation step, a clone of the epithelium model can be defined, having the current state of the grid as initial condition.

The reader is invited to consult the **documentation** available at EpiLog web site to get further information ( http://epilog-tool.org/documentation).

## Use cases

**Figure 2.**
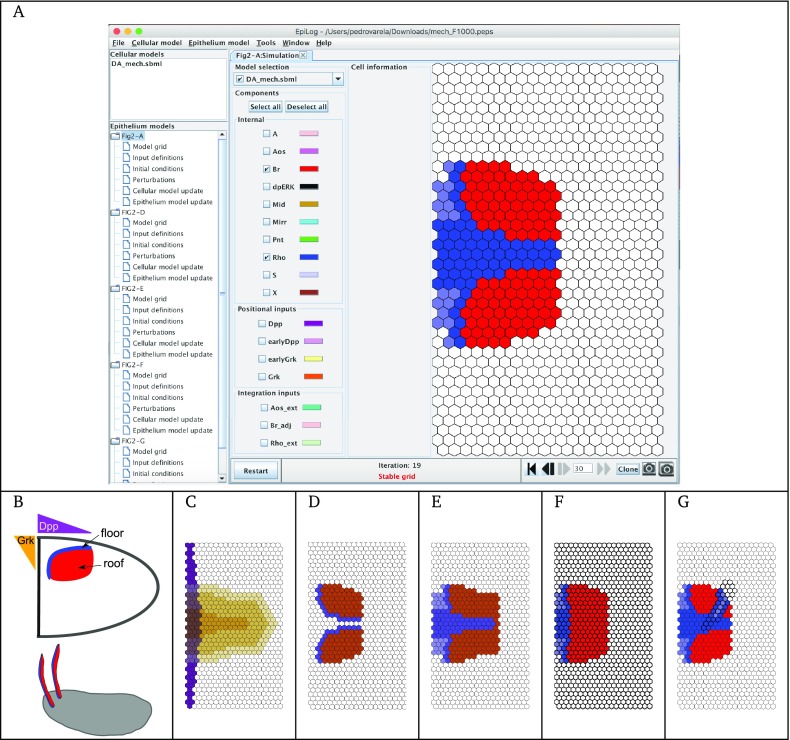
Model of the fly eggshell patterning of Fauré *et al.* ^5^, implemented in EpiLog. ( **A**) EpiLog main window with the stable pattern reached by a simulation starting with all the cells of the grid having their internal components at 0, and positional inputs defined as shown in panel **C** (phase 1). ( **B**) Egg chamber with two dorsal respiratory appendages (DA); DA primordia are established as 2 regions on both sides of the oocyte midline, with follicle cells expressing Broad (Br in red), future roof of the DA, and cells expressing Rhomboid (Rho in blue), future floor of the DA. Establishment of these regions involve Gurken signalling (Grk, in orange) and Decapentaplegic signalling (Dpp, in violet). ( **C**) Grk (in graded orange, with 3 different levels) and Dpp (in purple) gradient defined as positional inputs in EpiLog. ( **D**) Stable pattern obtained with a simulation starting from the pattern displayed in panel **A** and without the Grk signal (phase 2), suggesting that Grk extinction is required to split the floor regions (see 5). ( **E**) EpiLog simulation of a mild overexpression of Dpp. ( **F**) EpiLog simulation of Pointed (Pnt, internal component) loss-of-function. ( **G**) EpiLog simulation of Pnt gain-of-function clones. Note that in panels **F**– **G**, perturbed cellular models are indicated by bold borders in the grid.

As a first illustration, we consider a very simple model, in which the cellular model includes two markers. When considering a juxtacrine signal ( *i.e.*, only contacting cells can communicate), this model accounts for the well-known Delta-Notch signalling, which implements a lateral inhibition process (see e.g. 20). The model is provided in Supplementary Model File 1. In Figure 1A, a two-cell model is shown, to illustrate interactions within and between the cells. Remaining panels of Figure 1 show EpiLog simulation results, illustrating the different patterns obtained when varying the updating mode, the integration rules and the border conditions. The development of EpiLog was originally motivated by our second illustration, which relates to the dorsal appendage-forming regions of the *Drosophila Melanogaster* eggshell ^21^ (see Figure 2). The logical model defined by Fauré *et al.* reproduces the patterning of the anterior follicular epithelium of the oocyte, defining the floor (Rho expressing cells) and roof (Br expressing cells) of the future appendages ^5^. EpiLog window is shown in Figure 2A, with the simulation panel displaying the stable pattern obtained with this model. Interestingly, the model shows that to get split regions (as displayed in Figure 2C), Gurken signal emitted by the nucleus, and defined as positional input in the model ( Figure 2B), must be removed. This is simulated in a second phase, which is performed by cloning the model when reaching the stable pattern ( Figure 2A), and by setting the Gurken signal to zero all over the grid. The remaining panels E-G show different states of the grid, recapitulating patterns resulting from model perturbations. For further detail on this modelling study, the reader is referred to 5.

## Summary

To the best of our knowledge, EpiLog is the first software tool for the definition, simulation and visualisation of qualitative, logical (Boolean and multivalued) models over hexagonal grids. It provides a graphical user interface and tools to conveniently support the study of epithelial pattern formation, relying on a logical framework.

EpiLog is free, open source and implemented in Java for operating system independence. It relies on the SBML qual standard format to import cellular models.

## Software availability

EpiLog v1.1.1 is available from: http://epilog-tool.org/downloads

Source code available from: https://github.com/epilog-tool/epilog

Archived source code as at time of publication: https://doi.org/10.5281/zenodo.1320503 ^22^

License: GNU General Public License v3.0

## Author information

PV and CVR developed the software. PTM acquired funding, developed the software and supervised the project. CC designed the project, acquired funding and supervised the project. PLV, CVR, PTM and CC wrote the article.

## Supplementary material

**Supplementary File 1: Dynamics of the lateral inhibition model under synchronous and asynchronous updates.**

**Supplementary File 2: Dynamics of the lateral inhibition model under α-asynchronous updates.**

**Supplementary Movie 1: Oscillatory behaviour of the lateral inhibition model under a synchronous update (see Figure 1 panel B).**

**Supplementary Movie 2: Stable pattern of the lateral inhibition model under an α-asynchronous update (see Figure 1 panel C).**

**Supplementary Model File 1: EpiLog file including variations of the lateral inhibition model as illustrated in Figure 1.**

**Supplementary Model File 2: EpiLog file including variations of the DA model as illustrated in Figure 2.**
