# Supplementary material for: EpiLog: A software for the logical modelling of epithelial dynamics
Source: F1000Res. 2019 Mar 11;7:1145. Originally published 2018 Jul 27. [Version 2] doi: 10.12688/f1000research.15613.2 (PMC6173114; doi:10.12688/f1000research.15613.2)
Supplement: Supplementary file 1 [file f1000research-7-20225-s0000.tgz › 7b9feea7-8df4-45c1-ba12-7ecaaa87efcd_Supplementary_File_1_v2.pdf]

Supplementary text 1

Dynamics of the lateral inhibition model under synchronous and asynchronous updates

The 2 cell model as displayed in panel (A) below is a simple positive regulatory circuit with 4 regulatory components (two *Green* and 2 *Red*), 2 activations (denoted by normal arrows) and 2 inhibitions (denoted by blunt arrows). The behaviour of such an elementary circuit has been fully characterised by Remy *et al.* [1]. The synchronous and asynchronous State Transition Graphs of this model are shown below. The two stable states are coloured depending on which *Green* and *Red* components are active in cells 1 and 2 (same colour code as in Fig. 1, main text). Starting from a ”naive” state where all the components are set to 0, the synchronous dynamics is trapped in a terminal cycle (panel (B)), whereas the asynchronous dynamics ends up in one of the two stable states (panel (C)). When considered over a 2D grid of cells, these behaviours are reproduced, leading to oscillations under a synchronous update (Fig. 1-B, main text) or to a stable pattern when cells are updated asynchronously enough (Fig. 1, panels C-F, main text).

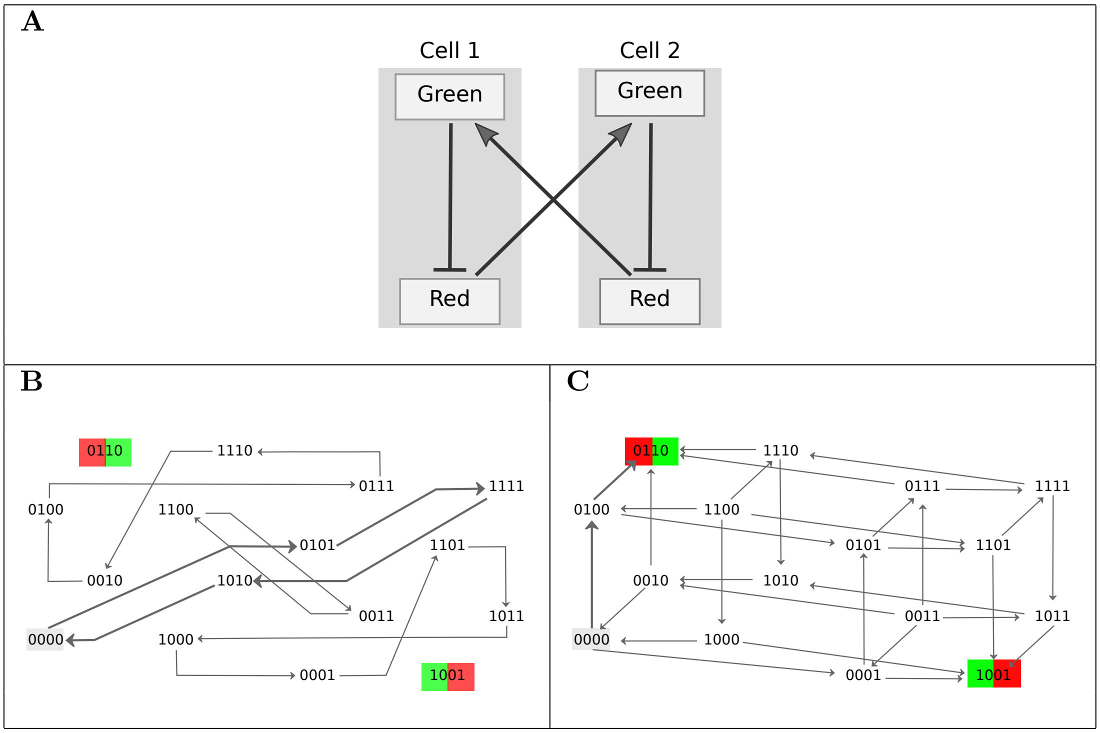

[1] E Remy, B Mossé, C Chaouiya, and D Thieffry. A description of dynamical graphs associated to elementary regulatory circuits. *Bioinformatics*, 19 Suppl 2:ii172–8, Oct 2003.
