# Supplementary material for: EpiLog: A software for the logical modelling of epithelial dynamics
Source: F1000Res. 2019 Mar 11;7:1145. Originally published 2018 Jul 27. [Version 2] doi: 10.12688/f1000research.15613.2 (PMC6173114; doi:10.12688/f1000research.15613.2)
Supplement: Supplementary file 2 [file f1000research-7-20225-s0001.tgz › b21082a1-0453-47ce-9c58-77164bcf35a2_Supplementary_File_2.pdf]

Supplementary text 2

Dynamics of the lateral inhibition model under α-asynchronous updates

Classical synchronous update of cellular automata can lead to unrealistic behaviours, as in the case of a grid of cells governed by a lateral inhibition model, which would synchronously alternate between two patterns (see Fig. 1 in the main text). The α-asynchrony aims to address this problem through the usage of an α parameter representing the proportion of cells called to effectively update at each step, effectively breaking the update synchrony of the cells. Originally, this method has been designed as assigning cells with a probability α for update [1].

EpiLog allows to choose a fixed simulation seed, ensuring that repeated simulations follow the same trajectories, *i.e.*, generate the same final outcome. Alternatively, the user can choose a random seed. It is also possible to specify if the sample proportion of cells to be updated comes from the set of cells called to update, or from the whole set of cells.

Table 1 below presents grids obtained through different values of the α parameter, starting from a naïve grid state (*i.e.* all cellular components at 0). For each α value, an intermediate and a final grid are presented.

Considering an α of 0 (asynchronous), a single cell is updated at each step. One can observe that after 400 steps, some cells are still in the naïve state, and that it takes 900 steps to get a stabilised grid.

Changing the α to 0.01, the equivalent intermediate grid state takes only 70 steps and only 370 steps are needed for stabilisation.

An α of 0.5, where 50% of the cells are called to update at each step, stabilisation occurs after only 12 steps.

Interestingly, increasing further the α to 0.8 the grid takes longer to stabilise. With a further increase to 0.99, stabilisation requires 132 steps. This is explained by the nature of the lateral inhibition model, and by the increasing number of updated cells as α increases. For the 0.99 case, 99% of the cells are called to update, which will cause almost the whole grid to alternate its state close to a purely synchronous behaviour.

Reference

[1] Nazim Fatès. Asynchronous cellular automata. In Robert A. Meyers, editor, *Encyclopedia of Complexity and Systems Science*, pages 1–21, Berlin, Heidelberg, 2018. Springer Berlin Heidelberg.

α	Steps	Transient grid	Steps	Final grid
0	400	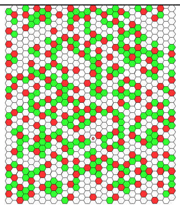	900	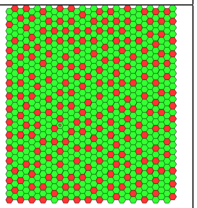
0.01	70	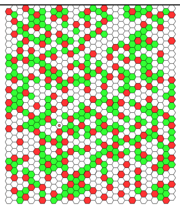	370	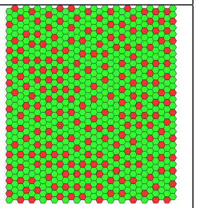
0.5	4	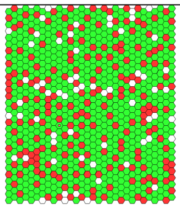	12	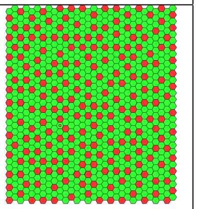
0.8	6	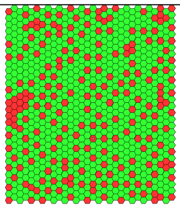	20	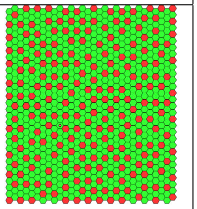
0.99	11	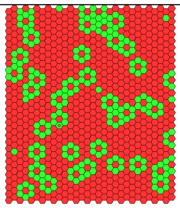	132	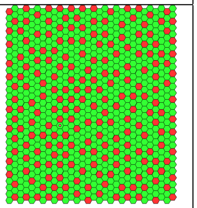

Table 1: Illustration of the impact of different values of α in α-asynchronous simulations of the lateral inhibition model (see Fig. 1 in the main text). Each table row displays a transient state and a stable state of the grid, for specific values of α. All simulations started from a naive grid configuration.
