# Supplementary material for: EpiLog: A software for the logical modelling of epithelial dynamics
Source: F1000Res. 2019 Mar 11;7:1145. Originally published 2018 Jul 27. [Version 2] doi: 10.12688/f1000research.15613.2 (PMC6173114; doi:10.12688/f1000research.15613.2)
Supplement: Peer review history

# Peer review: Reviewer response for version 2

**DOI:** 10.5256/f1000research.20225.r45550

**Published:** 2019-03-18

**Authors:** Arnau Montagud

**Affiliations:** 1 Institut Curie, Inserm U900, Mines Paris Tech, PSL Research University, Paris, France; 2 Barcelona Supercomputing Center (BSC), Barcelona, Spain

The authors have have done a good work in expanding and correcting all needed clarifications. I approve this improved version of the article.

I have read this submission. I believe that I have an appropriate level of expertise to confirm that it is of an acceptable scientific standard.

# Peer review: Reviewer response for version 1

**DOI:** 10.5256/f1000research.17032.r37601

**Published:** 2018-10-10

**Authors:** Arnau Montagud

**Affiliations:** 1 Institut Curie, Inserm U900, Mines Paris Tech, PSL Research University, Paris, France; 2 Barcelona Supercomputing Center (BSC), Barcelona, Spain

The paper describes a software used for qualitative simulations of logical models in a hexagonal grid, called EpiLog. The paper is well written, even though some parts could be slightly expanded for a better comprehension. The software is easy to use for people with logical modelling background and is a visual way of exploring populations of Boolean models and their effect on neighbouring cells. The software is freely available, cross-platform, can use open standards of logical models and its code can be studied and forked in GitHub.

 Nevertheless, the paper would benefit from some additions and clarifications:

 Major:

1. Expansion of introduction: The last sentence of first paragraph could be completed with some of the reasons cellular automaton are well suited. Also, the relationship of present paper and [1] could be better worded. It is unclear if authors used Mendes *et al *approach as is or if they improved Mendes *et al* approach and can work with more than a dozen cells. Addressing these would enhance the understanding of the work.
2. Discussion on the alpha parameter: The paper would benefit from a discussion on the use of “alpha-asynchronism” as logical modelling tools, until now, either used asynchronous and synchronous updates. Also, the paper presents results using alpha=1 (pure synchronous) or 0.25, but does not present results with alpha=0 (pure asynchronous). Presenting and discussing these (even in Supplementary) would allow users to have a better understanding on the effects of this parameter.
3. Detailed tutorial: Even though the Documentation does a good job detailing the possibilities of the tool, the software users would benefit from a detailed discussion on the effect of changing spatial initial conditions, alpha parameter and, in general, the biological relevance of these. This supplementary file would also allow to expand on the discussion of Figure 2 that is absent from the paper (understandably being a software tool paper).

Minor:

1. Comparison to other software: Even though this software is the first that includes hexagonal grid and Boolean models, naming some other software that can be used for similar projects (with their pros and cons) would give the reader a better sense on the current status of the field.
2. In Methods/Operation/Simulation settings, first sentence, authors say “For each epithelium, simulation settings define the updating schemes for both the cellular and epithelium models.” Does cellular model mean one cell and epithelium model the whole set of cells? As the phrase starts with epithelium, the current wording is a bit confusing.
3. Figure 1 legend: “Stable pattern” is a bit confusing, maybe it would be better to use “pattern of stable states”.
4. Figure 2 legend: Authors should describe “phase 1” and “phase 2” in the paper to understand what is the difference in panels C and D. In panel B, “DA” should be shown in the figure. In panel C, the colours for Grk and Dpp should be explained (even if they are described in previous panel).
5. In Supplementary text, in the last part of the last sentence, the Figure in main text you refer to is Figure 2, panels C-F.

Following previous reviewers’ comments, I had no errors using EpiLog on Windows.

I have read this submission. I believe that I have an appropriate level of expertise to confirm that it is of an acceptable scientific standard, however I have significant reservations, as outlined above.

## References

[1] MendesNDLangFLe CornecYSMateescuRBattGChaouiyaC: Composition and abstraction of logical regulatory modules: application to multicellular systems. *Bioinformatics*.2013;29(6) : 10.1093/bioinformatics/btt033 749-57 10.1093/bioinformatics/btt033 23341501

# Response

**Published:** 2019-02-28

**Authors:** Claudine Chaouiya

**Affiliations:** IGC, Portugal

We thank you for the detailed comments and suggestions. We have revised the manuscript accordingly, and we briefly answer your points below.

 We have revised the introduction, noting that cellular automata are a natural modelling choice to define local rules governing the behaviours of cells over a lattice. We also clarified the link between the framework as implemented in EpiLog and the work presented in Mendes et al 2013.

 We have included a supplementary text illustrating and discussing the use of the alpha-asynchronous update, and varying the values of alpha. This is done using the simple model of lateral inhibition. It is worth to note that a general statement of how the value of alpha impacts the dynamics (intermediate and final patterns) is hard to make, as such properties certainly depend on the model. Finally, alpha-asynchronism has been considered for cellular automata. The reviewer is right when noting that this update has not been employed for (cellular) logical models but, besides a theoretical interest, it is unclear if such an update would have any biological significance besides the fact that indeed a few cells may update their states simultaneously.

 Concerning your point on a detailed tutorial, in addition to the documentation,we now provide some video to illustrate the major steps in defining and simulating a model with EpiLog (available at https://www.youtube. com/channel/UCGGcpepqyYJkt7dhrhncQiQ, as indicated in the documentation). Results displayed in Figure 2 are thoroughly discussed in the original paper, Fauré et al. 2014.

 We now mention the two other software tools we are aware of that consider logical models in a multi-scale setting.

 We have clarified the terminology. In particular we have changed the legend of Fig 1, which now says ”stable state of the grid, referred to as stable pattern”. Indeed a stable pattern is defined by stable states of the cells, but not any combination of such cellular stable states remain stable in the multi-cellular model.

 Concerning Fig. 2, the requirement for the 2 phases is now shortly described in the main text. The legend has been modified as suggested, and dorsal appendage (DA) has been added in panel B.

# Peer review: Reviewer response for version 1

**DOI:** 10.5256/f1000research.17032.r38246

**Published:** 2018-10-04

**Authors:** Alejandro F. Villaverde, David Henriques

**Affiliations:** 1 Bioprocess Engineering Group, IIM-CSIC, Vigo, Spain; 2 Bioprocess Engineering Group, IIM-CSIC, Vigo, Spain

This paper describes a new software tool for modelling epithelial dynamics in 2D. The software, called EpiLog, uses a logical framework that assigns discrete values to the model variables. The approach used for specifying the logical rules was previously presented in a paper from the same senior author (Mendes et al ^1^).

 The paper is well written, and the software is provided in a nice dedicated website, which also includes documentation and additional information. Furthermore, the source code is provided at GitHub.

 We have a few comments and suggestions regarding both the methodological approach and the software implementation:

 In regard to the method:

 1) It is stated that the published method can only handle up to a dozen cells. However, the graphical interface seems to describe more. Maybe the authors could elaborate on the limitations of the tool, as well as on its performance for different types of problems.

 2) It is not totally clear whether the approach used in this paper is exactly the same as the one published in (Mendes et al ^1^). If it is not, and if there is no other published paper describing the method, an upgraded methods paper could be of interest. On the other hand, if there is another publication describing the method, it should be cited in the paper. It is also unclear if the method has been previously validated in epithelium (hexagonal) case. The authors may want to clarify these points in a revised version.

 3) We are aware that, being a software tool article, the present paper is probably not the best place for extended discussions about biological aspects. The following points may therefore be considered as suggestions for the content of an upgraded paper as described in the previous paragraph. Of course, they would also be welcome additions to an extended version of the present paper, if the authors deem them appropriate to address:

 3.1) It may be useful to describe how to define a new (toy) model and a grid from scratch.

 3.2) What type of cues/signals are considered?

 3.3) A real case study would make it easier to follow the pipeline.

 3.4) Some additional biological explanation or motivation of potential applications might make the paper more attractive.

 In regard to the software:

 1) We tested EpiLog in Windows. We managed to load and simulate the Drosophila "Eggshell Patterning", but the software reported warnings/errors. Are these relevant?

We have read this submission. We believe that we have an appropriate level of expertise to confirm that it is of an acceptable scientific standard.

## References

[1] MendesNDLangFLe CornecYSMateescuRBattGChaouiyaC: Composition and abstraction of logical regulatory modules: application to multicellular systems. *Bioinformatics*.2013;29(6) : 10.1093/bioinformatics/btt033 749-57 10.1093/bioinformatics/btt033 23341501

# Response

**Published:** 2019-02-28

**Authors:** Claudine Chaouiya

**Affiliations:** IGC, Portugal

We thank you for the comments and suggestions, and we briefly answer each point or the report below.

 1) We believe the misunderstanding was due to the confusion with our mention to a previous work (Mendes at al 2013). This should now be clarified.

 2) This should be clear in the revised text. The rationale to define cell-cell communication (i.e. the rules governing the behaviours of input components of cellular models) relies on the definitions presented in Mendes et al 2013. However, EpiLog provides a graphical 1 interface and a simulation platform, producing a single model trajectory, whereas Mendes et al. aimed at determining all the stable states reachable from a given initial condition and considering an asynchronous update.

 3) 3.1 should now be addressed through the video that are available on EpiLog web site

 3.2 integration inputs are now more explicit in the text (juxtacrine signals, or other secreted molecules influencing cells in the neighbourhood)

 3.3 the DA formation is a real case study. The interested reader can refer to the original paper by Fauré et al., and can also download the model from EpiLog web site.

 3.4 Indeed, we intend to include further arguments on biological applications in a forthcoming paper on lattice-based modelling approaches

 4) Regarding the software: EpiLog relies on bioLQM, which in turn relies on JSBML libraries. The warning messages are being thrown by JSBML when loading a cellular model, but do not affect any of EpiLog’s behaviour.

# Peer review: Reviewer response for version 1

**DOI:** 10.5256/f1000research.17032.r37297

**Published:** 2018-08-20

**Authors:** Stefano Nichele

**Affiliations:** 1 Department of Computer Science, Oslo Metropolitan University, Oslo, Norway

The article describes EpiLog, a software tool to model artificial development of epitelial tissue through cellular automata with hexagonal cells. 

 EpiLog provides a high degree of customization through the graphical user interface. This includes: (a) model definition, such as CA size, logical models and initial states; (b) simulation settings, including updates (synchronous or asynchronous/probabilistic) and (c) perturbations on single cells or regions. 

 The article provides two simple use cases to introduce the user to the software. First, a model of lateral inhibition process is presented in FIg. 1. Second, a developmental model of Drosophila eggshell. 

 Minor revisions:

 The article would benefit by the following:

- background section with related work and description of CA/artificial development software tools, with brief description of similarities and differences;
- a more advanced use case described in details (for the advanced reader).

I have read this submission. I believe that I have an appropriate level of expertise to confirm that it is of an acceptable scientific standard.

# Response

**Published:** 2019-02-28

**Authors:** Claudine Chaouiya

**Affiliations:** IGC, Portugal

We than you for the suggestions.  We considered that a proper state of the art was beyond the scope of this application note. However, we now acknowledge the existence of two closely related tools, CoGNaC and PhysiBoSS.  The DA formation is a real, advanced case study. The interested reader can refer to the original paper by Fauré et al., and can also download the model from EpiLog web site.
